# A New Child-Turcotte-Pugh Class 0 for Patients with Hepatocellular Carcinoma: Determinants, Prognostic Impact and Ability to Improve the Current Staging Systems

**DOI:** 10.1371/journal.pone.0099115

**Published:** 2014-06-06

**Authors:** Yun-Hsuan Lee, Chia-Yang Hsu, Chen-Wei Chu, Po-Hong Liu, Cheng-Yuan Hsia, Yi-Hsiang Huang, Chien-Wei Su, Yi-You Chiou, Han-Chieh Lin, Teh-Ia Huo

**Affiliations:** 1 Faculty of Medicine, National Yang-Ming University School of Medicine, Taipei, Taiwan; 2 Institute of Clinical Medicine, National Yang-Ming University School of Medicine, Taipei, Taiwan; 3 Institute of Pharmacology, National Yang-Ming University School of Medicine, Taipei, Taiwan; 4 Department of Medicine, Taipei Veterans General Hospital, Taipei, Taiwan; 5 Department of Surgery, Taipei Veterans General Hospital, Taipei, Taiwan; 6 Department of Radiology, Taipei Veterans General Hospital, Taipei, Taiwan; 7 Department of Medicine, Yuanshan Branch of Taipei Veterans General Hospital, Taipei, Taiwan; 8 Department of Biostatistics, University of California Los Angeles, Los Angeles, California, United States of America; Icahn School of Medicine at Mount Sinai, United States of America

## Abstract

**Background/Aim:**

Majority of patients with hepatocellular carcinoma (HCC) belonged to Child-Turcotte-Pugh (CTP) class A. We aimed to identify a new class of patients with very well-preserved liver function and analyze its impact on outcome prediction, tumor staging and treatment allocation.

**Methods:**

A total of 2654 HCC patients were retrospectively analyzed. The prognostic ability was compared by the Akaike information criterion (AIC).

**Results:**

The CTP class 0 was defined by fulfilling all criteria of albumin ≧4 g/dL, bilirubin ≦0.8 mg/dL, prothrombin time prolongation <0 seconds, no ascites and encephalopathy. A total of 23% of patients of CTP class A were reclassified as CTP class 0. Patients with CTP class 0 had a higher serum sodium level, lower serum creatinine, alanine aminotransferase, α-fetoprotein levels, shorter prothrombin time, better general well-being, smaller tumor burden with more solitary nodules, lower rates of vascular invasion, ascites formation, hepatic encephalopathy, more frequently treated with curative interventions and better Barcelona Clinic Liver Cancer (BCLC) stages (all p<0.001). In the Cox proportional hazards model, the adjusted hazard ratios for CTP class A, B and C were 1.739, 3.120 and 5.107, respectively, compared to class 0 (all p<0.001). Reassigning patients with CTP class 0, A, B, B and C to stage 0, A, B, C and D, respectively, provided the lowest AIC score among all BCLC-based models.

**Conclusions:**

The proposal of CTP class 0 independently predicted better survival in HCC patients. Modification of tumor staging systems according to the modified CTP classification further enhances their prognostic ability.

## Introduction

Hepatocellular carcinoma (HCC) is a common malignancy accounting for 500,000 deaths per year [1]. Chronic inflammation of liver parenchyma, mainly arising from viral hepatitis or alcoholism, may subsequently result in HCC. Notably, majority of patients with HCC have a coexisting liver cirrhosis or chronic liver disease. The Child-Turcotte-Pugh (CTP) classification was originally created to predict mortality in patients receiving surgery for portal hypertension [2], [3], and is currently used to determine the survival of patients with chronic liver disease and cirrhosis. Among the commonly used cancer staging systems, CTP classification is an important outcome indicator in the Barcelona Clinic Liver Cancer (BCLC), Cancer of the Liver Italian Program (CLIP) and Taipei Integrated Scoring (TIS) staging systems [4]–[7]. CTP classification is also included as an important index for treatment suggestion, according to the BCLC staging system [7], [8].

The current CTP classification is determined by serum albumin, bilirubin and international normalized ratio (INR) of prothrombin time (PT) levels, ascites formation and encephalopathy. Interestingly, even though CTP class A comprises only patients with total scores of 5 and 6, it represents a majority of patients with HCC [9]. Furthermore, the composition of HCC patients with CTP score of 5 encompasses different strata of clinical scenarios, including patients with no chronic liver disease, patients with only chronic inflammation and patients with well-compensated cirrhosis. The long-term outcomes of these patients are considered to be different [10], [11] and the current CTP classification may not be capable of distinguishing these patients in estimating liver function and survival prediction. Moreover, the current cancer staging system, based on the original CTP classification, may not be informative enough [12]–[14]. A new definition for an earlier stage of CTP classification is considered for better discrimination of liver functional reserve. No study to date has specifically aimed to explore the rationale of using the CTP classification with an attempt to identify the early stage of these patients. In this study, we proposed a new CTP class 0 and validated its ability in predicting outcome for HCC. Moreover, modified models of the BCLC, CLIP and TIS staging systems were proposed according to the modified CTP classification, and the prognostic ability of these models were investigated.

## Patients and Methods

### Patients

Between 2002 and 2012, patients who were newly diagnosed with HCC in Taipei Veterans General Hospital were consecutively enrolled and retrospectively analyzed. The baseline information was comprehensively collected at the time of diagnosis. This study has been approved by the institutional review board of Taipei Veterans General Hospital and complies with the standards of the Declaration of Helsinki and current ethical guidelines. Waiver of consent was obtained, and patient records/information was anonymized and de-identified prior to analysis. Part of our patients had been enrolled in our previous studies [9], [15].

### Diagnosis and Definitions

The inclusion criteria were: (1) patients with newly diagnosed HCC; (2) aged above 18 years; (3) did not receive management for HCC before enrollment. Patients who were diagnosed with malignant tumor other than HCC were excluded from the study. The diagnosis of HCC was established by histology or based on the findings of typical radiological features in a 4-phase multi-detector computed tomography scan or dynamic contrast-enhanced magnetic resonance imaging [8], [16], [17]. Vascular invasion was defined by the presence of adjacent thrombus to the tumor in portal vein with blurring boundary confirmed by at least one imaging modality. Alcoholism was diagnosed in subjects with a documented history of alcohol excess of at least 40 g alcohol daily for 5 years or more [18]. The performance status was assessed by using the Eastern Cooperative Oncology Group (ECOG) performance scale: 0 (asymptomatic) to 4 (confined to bed). The original CTP classification and the model for end-stage liver disease (MELD) score were defined as reported [2], [3], [19]. Total tumor volume (TTV) was calculated as the sum of all tumor nodule volume, and each tumor nodule volume is calculated as 4/3×3.14×(maximum radius of the tumor nodule in cm)^3^ as previously described [6]. The BCLC, CLIP and TIS systems were used to define clinical staging [4]–[6].

### Treatment

Treatment for each individual patient was suggested by a multidisciplinary HCC team of our hospital. Curative treatment was defined as patients undergoing surgical resection, local ablation therapy or liver transplantation. Other treatment modalities were mostly palliative and collectively defined as non-curative treatment. Patients with HCC selected for surgical resection (SR) were (a) patients with tumor involving no more than 3 Healey’s segments, (b) patients with preserved liver function and had less than 25% retention of indocyanine green 15 minutes (ICG15) after injection [20], and (c) patients who had no main portal vein trunk involvement or distant metastasis [8], [17]. The ICG15 of each patient was then individually evaluated for the feasibility of SR according to their tumor size and the extent of hepatic resection. Radiofrequency ablation (RFA) was considered in patients with solitary tumor nodule ≤5 cm or up to 3 tumor nodules with sizes ≤3 cm and not suitable or unwilling to receive surgery [8], [17]. RFA was also considered as the alternative first-line treatment in patients with single solitary tumor nodule ≤2 cm [21].

### Proposal of a New CTP Class 0 and Modified BCLC, CLIP and TIS Models

In order to select new cutoff values for serum albumin, bilirubin and INR of PT for patients with very well-preserved liver function, the distribution of the study patients and clinical feasibility were taken into consideration. The cutoff values were set more strictly with a higher standard than the normal range of our center laboratory to identify patients without overt liver dysfunction. Modified models for tumor staging were proposed by re-defining the CTP class for each stage of the original BCLC, CLIP and TIS staging systems.

### Statistical Methods

The chi-squared test and the Kruskal-Wallis test were used to compare categorical and continuous data of more than 2 groups. The Kaplan-Meier method with a log-rank test was applied to compare the survival. For continuous variables, the median of each variable was used as the cutoff to dichotimize patients in the survival analysis. Factors which were significant (p<0.05) in the univariate analysis were introduced into multivariate Cox proportional hazards model. For all comparisons, a p value<0.05 was considered statistically significant.

The overall predictive accuracy of the survival between different tumor staging models was compared to determine which system possessed the most accurate prediction of survival (monotonicity of the score). Homogeneity (small difference in survival among patients in the same score category within each model) was determined by likelihood ratio χ^2^ which was generated by the Cox proportional hazards model [22], [23]. The consequences of the Cox regression were expressed with the Akaike information criterion (AIC), which revealed how the scoring systems affected the patient survival. The lower the AIC, the more explanatory and informative the model is [24].

## Results

### Definitions for the New CTP Class 0

The new CTP class 0 were defined by fulfilling all the criteria of serum albumin ≧4.0 g/dL, bilirubin ≤0.8 mg/dL, PT prolongation <0 second, no ascites and no encephalopathy (Table 1). A total of 917 patients (48% of original CTP class A) had a serum albumin level ≧4.0 g/dL, 1110 patients (58% of original CTP class A) had a serum bilirubin ≤0.8 mg/dL, 1454 patients (76% of original CTP class A) had a PT prolongation <0 second, and 1798 patients (94% of original CTP class A) did not have ascites. When combining these criteria together, 441 patients (23% of original CTP class A) fulfilled all the above mentioned criteria and were redefined as CTP class 0. Patients with CTP score of 5–6 and not fulfilling the criteria for CTP class 0 were classified as modified CTP class A; the definitions for CTP class B and C remained the same.

**Table 1:** Definitions for the original and modified CTP classifications.

	Child-Turcotte-Pugh Scoring				
	Albumin(g/dL)	Bilirubin(mg/dL)	Prothrombin timeprolong (seconds)	Ascites	Encephalopathy
1	>3.5	<2	<4	None	None
2	2.8–3.5	2–3	4–6	Mild	Grade 1–2
3	<2.8	>3	>6	Moderate to severe	Grade 3–4

Modified CTP class 0: Fulfill all criteria of albumin ≧4.0 g/dL, bilirubin ≦0.8 mg/dL, prothromin time prolongation <0 seconds, no ascites and no encephalopathy.

Modified CTP class A: total scores of 5–6 and not fulfilling criteria for class 0; class B: total scores of 7–9; class C: total scores of 10–15.

**Abbreviations**: CTP, Child-Turcotte-Pugh.

### Distribution of Patients after Modifying CTP Classification

A total of 2654 HCC patients were consecutively enrolled and analyzed in this study. There were a total of 1924 (72%), 583 (22%) and 147 (6%) patients in the original CTP class A, B and C, respectively (Table 2). After modifying the CTP classification, 441 (23%) patients in the original CTP class A were reassigned to the modified CTP class 0. A total of 441 (17%), 1483 (56%), 583 (22%) and 147 (6%) patients were in the modified CTP class 0, A, B and C, respectively.

**Table 2:** The distribution of HCC patients after modification of the CTP classifications.

		Modified Child-Turcotte-Pugh Classification				
		0	A	B	C	No. (%)
Original Child-Turcotte-Pugh Classification	A	441	1483	-	-	1924 (72)
	B	-	-	583	-	583 (22)
	C	-	-	-	147	147 (6)
	No. (%)	441 (17)	1483 (56)	583 (22)	147 (6)	

**Abbreviations**: CTP, Child-Turcotte-Pugh; HCC, hepatocellular carcinoma; No., number.

### Patient Characteristics

The baseline characteristics of all HCC patients and patients with modified CTP class 0, A, B and C are shown in Table 3. The study patients were predominantly male (77%) with a mean age of 64 years. The most common etiology for chronic liver disease is HBV only (42%). The mean and median CTP scores of all patients were 6.1 and 5, respectively, and the mean and median MELD scores were 9.8 and 8.3, respectively. Patients with CTP class 0 had a higher percentage of HBV infection in etiology, higher serum albumin and sodium levels, lower serum bilirubin, creatinine, INR of PT, alanine aminotransferase (ALT), lower serum α-fetoprotein (AFP) level, better general well-being, lower mean CTP and MELD scores, smaller tumor burden with more solitary nodules, lower rates of vascular invasion, ascites formation, hepatic encephalopathy, more frequently treated with curative interventions and better BCLC, CLIP and TIS stages (all p<0.001).

**Table 3:** Baseline demographics of all HCC patients and HCC patients with modified CTP class 0, A, B and C.

	All	Modified CTP class				p
		0	A	B	C	
Number of patients	2654	441	1483	583	147	
Age (years,mean±SD)	64±13	63±13	65±13	64±14	60±13	<0.001
Male/female (%)	77/23	81/19	76/24	77/23	80/20	0.22
Etiology of chronicliver disease(%)						<0.001
HBV only	1103(42)	213(48)	619(42)	209(36)	62(42)	
HCV only	609(23)	79(18)	384(26)	124(21)	22(15)	
HBV+HCV only	90(3)	15(3)	54(4)	17(3)	4(3)	
Alcohol only	121(5)	13(3)	59(4)	38(7)	11(8)	
Multiple etiologiesand others	731(28)	121(27)	367(25)	195(33)	48(33)	
Serum biochemistry(mean±SD)						
Albumin (g/dL)	3.7±0.6	4.3±0.2	3.8±0.4	3.1±0.5	2.6±0.5	<0.001
Bilirubin (mg/dL)	1.5±2.7	0.6±0.2	0.9±0.4	2.4±3.3	6.8±7.0	<0.001
BUN (mg/dL)	19±12	18±10	18±10	21±16	24±17	<0.001
Creatinine (mg/dL)	1.2±1.0	1.2±1.2	1.1±0.9	1.3±1.2	1.5±1.4	<0.001
INR of PT	1.09±0.18	0.99±0.07	1.05±0.09	1.17±0.20	1.46±0.34	<0.001
ALT (U/L)	73±92	54±47	71±87	81±92	110±187	<0.001
Sodium (mmol/L)	138±4	140±3	139±3	137±4	133±6	<0.001
AFP [ng/mL, mean±SD(median; IQR)]	26,155±241,302(49; 849)	5,852±37,162(21; 186)	20,628±288,840(43; 483)	46,144±198,882(111; 4768)	63,551±211,663(186; 4392)	<0.001
Performance status0/1/2/3/4 (%)	58/18/12/8/4	82/10/6/2/0	68/18/10/3/1	28/25/20/17/11	4/13/20/42/22	<0.001
Mean CTP score[mean±SD(median; IQR)]	6.1±1.6 (5; 2)	5.0±0 (5; 0)	5.4±0.5 (5; 1)	7.7±0.8 (7; 1)	10.9±1.0 (11; 1)	<0.001
MELD score						
<8/8–12/12–16/>16(%)	44/36/11/8	72/23/3/2	53/39/6/3	13/45/28/14	0/6/29/65	<0.001
Score [mean±SD(median; IQR)]	9.8±4.2 (8.3; 3.8)	7.8±2.4(7.1; 1.7)	8.6±2.6 (7.9; 2.3)	12.0±4.0(11.2; 4.8)	19.0±6.0(17.3; 6.1)	<0.001
No. and size of tumor (%)						
Single/multiple	60/40	69/31	61/39	56/44	44/56	<0.001
≤5 cm/>5 cm	56/44	66/34	59/41	41/59	46/54	<0.001
TTV [cm^3^, mean±SD(median; IQR)]	374±729(51; 392)	201±458(29; 139)	332±708(39; 291)	584±890(183; 773)	491±688(166; 679)	<0.001
Vascular invasion(%)	928(35)	106(24)	429(29)	307(53)	86(59)	<0.001
Ascites (%)	646(24)	0(0)	126(9)	380(65)	140(95)	<0.001
Encephalopathy (%)	83(3)	0(0)	0(0)	42(7)	41(28)	<0.001
Treatment modality(%)						<0.001
Resection	705(27)	198(45)	459(31)	46(8)	2(1)	
Transplantation	8(0.3)	1(0.2)	1(0.1)	1(0.2)	5(3)	
Local ablation	529(20)	97(22)	323(22)	91(16)	18(12)	
TACE	769(29)	117(27)	489(33)	144(25)	19(13)	
Sorafenib	169(6)	5(1)	62(4)	87(15)	15(10)	
Others	474(18)	23(5)	149(10)	214(37)	88(60)	
BCLC stage0/A/B/C/D (%)	9/22/14/42/14	13/31/19/35/2	11/24/17/43/5	0/13/7/54/27	0/0/0/0/100	<0.001
CLIP score0/1/2/3/4/5/6 (%)	27/26/16/12/12/6/2	46/27/14/8/5/0/0	34/30/15/11/10/0.1/0	0/21/21/14/20/24/0	0/0/14/20/18/20/27	<0.001
TIS score0/1/2/3/4/5/6 (%)	35/22/12/12/11/6/1	53/23/10/9/5/0/0	46/21/9/13/10/0/0	0/29/17/13/17/24/0	0/0/31/17/12/17/23	<0.001

**Abbreviations**: HCC, hepatocellular carcinoma; CTP, Child-Turcotte-Pugh; SD, standard deviation; HBV, hepatitis B virus; HCV, hepatitis C virus; BUN, blood urea nitrogen; INR, international normalized ratio; PT, prothrombin time; ALT, alanine aminotransferase; AFP, α-fetoprotein; IQR, interquartile range; MELD, model for end-stage liver disease; No., number; TTV, total tumor volume; TACE, transcatheter arterial chemoembolization; BCLC, Barcelona Clinic Liver Cancer; CLIP, Cancer of the Liver Italian Program; TIS, Taipei Integrated Scoring.

### Survival Analysis

**Figure 1:**
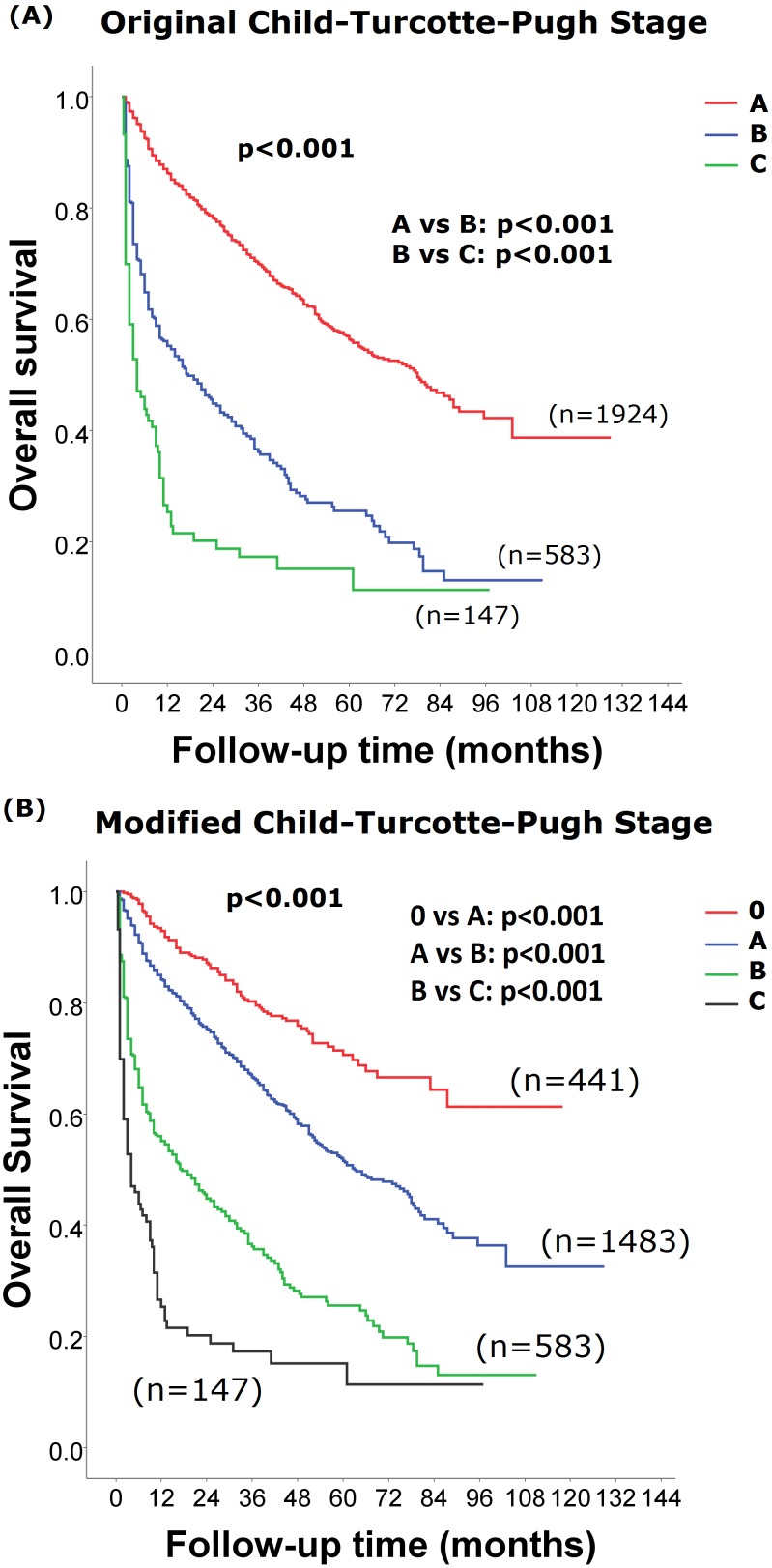
Comparison of survival distributions between patients of different CTP classifications. Patients with the new CTP class 0 were associated with a better long-term survival (panel B; p<0.001). Pairwise comparison between each CTP and modified CTP classes showed significant survival differences (panel A p<0.001).

During a median follow up period of 18 [range 0.5–129, interquartile range (IQR) 40] months, the cumulative 3- and 5- year survival of patients with original CTP class A, B and C were 70% and 50%, 36% and 26% and 17% and 15%, respectively (p<0.001; Fig. 1A).

The 3- and 5- year survival of patients with modified CTP class 0, A, B and C were 80% and 71%, 67% and 52%, 36% and 26% and 17% and 15%, respectively (p<0.001; Fig. 1B). Survival comparisons of other baseline characteristics are shown in Table 4. Patients with a high serum creatinine and AFP levels, low serum sodium level, large TTV, poor performance status, presence of vascular invasion and receiving non-curative treatments were associated with a worse long-term outcome in the univariate analysis (all p<0.001). In the Cox proportional hazards model, the adjusted hazard ratio for CTP class A, B and C were 1.739 [95% confidence interval (CI):1.395–2.168; p<0.001], 3.120 (CI:2.442–3.986; p<0.001) and 5.107 (CI:3.758–6.940; p<0.001), respectively, when compared to CTP class 0. Moreover, other independent prognostic predictors identified in the Cox model were serum creatinine and AFP levels, TTV, performance status, vascular invasion and receiving non-curative treatments (all p≤0.001).

**Table 4:** Univariate and multivariate Cox regression survival analysis in HCC patients.

		Univariate analysis			Multivariate analysis		
	N	3-year survival (%)	5-year survival (%)	p	HR	95% CI	p
Sex (male/female)	2054/600	61/63	48/50	0.283			
Age (≤65/>65 years)	1362/1292	61/61	52/46	0.254			
HBsAg (negative/positive)	1187/1467	61/61	46/51	0.54			
Creatinine (≤1.0/>1.0 mg/dL)	1563/1091	64/56	53/43	<0.001	1.226	1.079–1.392	0.002
Sodium (≤139/>139 mmol/L)	1513/1141	53/71	44/55	<0.001			
AFP (≤50/>50 ng/mL)	1333/1321	73/47	58/38	<0.001	1.679	1.469–1.919	<0.001
TTV (≤50/>50 cm^3^)	1325/1329	75/44	61/33	<0.001	1.544	1.326–1.798	<0.001
Performance status (ECOG 0/1–4)	1536/1118	74/38	61/19	<0.001	1.959	1.679–2.287	<0.001
Vascular invasion (No/Yes)	1726/928	70/39	56/34	<0.001	1.703	1.467–1.977	<0.001
Treatment(curative/non-curative)	1242/1412	78/42	65/30	<0.001	2.319	2.005–2.681	<0.001
Modified CTP class				<0.001			
0	441	80	71		1		
A	1483	67	52		1.739	1.395–2.168	<0.001
B	583	36	26		3.120	2.442–3.986	<0.001
C	147	17	15		5.107	3.758–6.940	<0.001

**Abbreviations**: HCC, hepatocellular carcinoma; HR, hazard ratio; CI, confidence interval; HBsAg, hepatitis B surface antigen; AFP, α-fetoprotein; TTV, total tumor volume; ECOG, Eastern Cooperative Oncology Group; CTP, Child-Turcotte-Pugh.

The long-term survival of different treatment strategies stratified by CTP class 0 and A were analyzed. In patients with CTP class 0, the 3-year survival rates for patients with SR, local ablation, TACE, sorafenib and other treatments were 84%, 88%, 73%, 50% and 36%, respectively (p<0.001). In patients with modified CTP class A, the 3-year survival rates for the corresponding groups were 80%, 76%, 57%, 22% and 26%, respectively (p<0.001).

### Proposals of Modified BCLC, CLIP and TIS Staging Systems Based on the New CTP Classification

The staging and scoring criteria were re-defined based on the new CTP classification in the 9 modified BCLC, CLIP and TIS models (Table 5&6). In modified BCLC model A, CTP class 0, A, A, B and C were assigned to stage 0-D, respectively. In modified BCLC model B, CTP class 0, A, B, B and C were assigned to stage 0-D, respectively. In modified BCLC model C, CTP class 0, A–B, B, B and C were assigned to stage 0-D, respectively. Criteria of other factors remained the same.

**Table 5:** The proposed criteria for original and modified models of the BCLC staging system.

Stage	BCLC	Modified BCLCModel A	Modified BCLCModel B	Modified BCLCModel C
0 (Very early)	Performance status (PS) 0; one small tumor ≤2 cm;			
	Original Child-Turcotte-Pugh (CTP) class A	Modified CTP class 0		
A (Early)	PS 0; single tumor ≤5 cm or 3 or few nodules ≤3 cm;			
	Original CTP class A–B	Modified CTP class A	Modified CTP class A	Modified CTP class A–B
B (Intermediate)	PS 0; large/multiple HCC;			
	Original CTP class A–B	Modified CTP class A	Modified CTP class B	Modified CTP class B
C (Advanced)	PS 1–2; vascular invasion or extrahepatic spread;			
	Original CTP class A–B	Modified CTP class B		
D (End stage)	PS 3–4; any tumor burden;			
	Original CTP class C	Modified CTP class C		

Original BCLC stage 0, A and B: all criteria should be fulfilled; stage C and D: at least one criterion should be fulfilled.

Modified BCLC stage 0: all criteria should be fulfilled; stage A, B, C and D: at least one criterion should be fulfilled.

**Abbreviations**: CTP, Child-Turcotte-Pugh; BCLC, Barcelona Clinic Liver Cancer; PS, performance status.

**Table 6:** The proposed criteria for original and modified models of the CLIP and TIS staging systems.

	Prognostic predictors for hepatocellular carcinoma patients								Scores
	Tumoral factor		Liver function				Tumor behavior		
	Total tumorvolume	Tumor numberand size	Original Child-Turcotte-Pugh (CTP) classification	Modified CTPclassification			α-fetoprotein	Vascularinvasion	
	<50/50–250/250–500/>500	Single and <50% liver span/multipleand <50% liver span/≧50% liver span	A/B/C	0/A/B/C	0/A/B-C	0/A–B/C	<400/>400	Absence/presence	
Scores	0/1/2/3	0/1/2	0/1/2	0/1/2/3	0/1/2	0/1/2	0/1	0/1	
**CLIP**		V	V				V	V	0–6
**Modified CLIP Model A**		V		V			V	V	0–7
**Modified CLIP Model B**		V			V		V	V	0–6
**Modified CLIP Model C**		V				V	V	V	0–6
**TIS**	V		V				V		0–6
**Modified** **TIS** **Model A**	V			V			V		0–7
**Modified** **TIS** **Model B**	V				V		V		0–6
**Modified** **TIS** **Model C**	V					V			0–6

**Abbreviations**: CTP, Child-Turcotte-Pugh; CLIP, Cancer of the Liver Italian Program; TIS, Taipei Integrated Scoring.

In the modified CLIP and TIS model A patients, scores of 0, 1, 2 and 3 were assigned to CTP class 0, A, B and C, respectively. Scores of 0, 1 and 2 were assigned to patients with CTP class 0, A and B–C and class 0, A–B and C, respectively, in patients with modified CLIP and TIS model B and C patients. Scores for other factors remained the same.

### Comparison of Prognostic Ability of the Original and Modified BCLC, CLIP and TIS Systems

The distributions of patients with different stages of the original and modified BCLC, CLIP and TIS staging systems are shown in Table 7. Among the 4 BCLC-based staging systems, the modified model B showed the lowest AIC value, followed by the modified model C and the modified model A, and lastly, the original system. Among the 4 CLIP-based staging systems and the 4 TIS-based staging systems, the modified model A had the best prognostic ability, followed by the modified model B, the original systems, and lastly, the modified model C. Among all 12 original and modified staging systems, the modified CLIP model A had the lowest AIC value.

**Table 7:** Comparison of the prognostic ability among 4 BCLC-, 4 CLIP- and 4 TIS-based staging systems.

N = 2654	Distribution ofpatients (%)	Homogeneity(Likelihood ratio χ^2^)	Akaikeinformation criterion
BCLC models (0/A/B/C/D)			
Original	9/22/14/42/14	492.449	13695.982
Modified A	2/25/13/46/14	500.669	13687.762
Modified B	2/25/17/42/14	518.292	13670.139
Modified C	2/28/14/42/14	502.622	13685.809
CLIP models [0/1/2/3/4/5/6(/7)]			
Original	27/26/16/12/12/6/2	710.818	13477.613
Modified A	8/24/24/15/12/11/6/2	762.847	13425.584
Modified B	8/24/25/15/11/11/7	714.275	13474.156
Modified C	8/28/25/14/13/12/2	622.205	13566.226
TIS models [0/1/2/3/4/5/6(/7)]			
Original	35/22/12/12/11/6/1	685.371	13503.060
Modified A	9/30/20/12/12/10/6/1	745.476	13442.955
Modified B	9/30/21/11/12/11/7	691.347	13497.084
Modified C	9/36/19/10/13/12/1	595.013	13593.418

**Abbreviations**: BCLC, Barcelona Clinic Liver Cancer; CLIP, Cancer of the Liver Italian Program; TIS, Taipei Integrated Scoring.

### Long-term Survival among Patients with Different Stages of BCLC, CLIP and TIS

**Figure 2:**
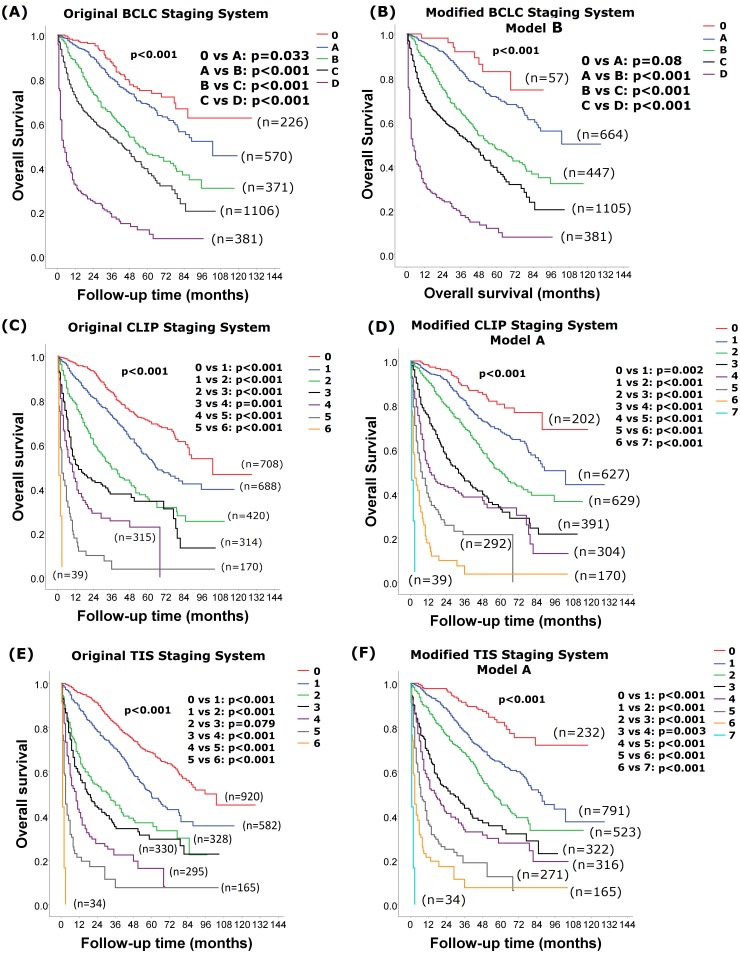
Comparison of survival between stages in the original and modified models BCLC, CLIP and TIS with the lowest AIC score. Patients with a more advanced stage were associated with a worse long-term survival in the original (panel A) and modified model B (panel B) BCLC systems, the original (panel C) and modified model A (panel D) CLIP systems, the original (panel E) and modified model A (panel F) TIS systems (all p<0.001). Pairwise comparison of survival differences between all stages in the modified BCLC, CLIP and TIS staging systems showed significant differences between each stage (all p<0.05), except for patients with modified BCLC stage 0 and A (p = 0.08).

The survival distributions among patients with different stages of original and modified BCLC, CLIP and TIS models with the lowest AIC scores are shown in Fig. 2A–2F. Patients with a more advanced stage were associated with a worse long-term survival in the original and modified model B BCLC systems, the original and modified model A CLIP systems, the original and modified model A TIS systems (Fig. 2A–2F; all p<0.001).

## Discussion

The CTP classification has been used to estimate liver functional reserve and predict survival among chronic liver disease patients. In our study, a total of 1924 (72%) patients belonged to the original CTP class A. The lack of the ability to discriminate liver function and outcome among patients with well-preserved liver function is a major drawback of the original CTP classification. The proposal of the new CTP class 0 in the modified CTP classification, which is aimed to more specifically define early stage patients, showed more accurate outcome prediction for HCC patients. In the modified CTP classification, 441 (23%) of patients with very-well preserved liver function in the original CTP class A were re-classified as CTP class 0. Notably, CTP class 0 was associated with a better long-term survival, with a 74% to 4-fold decrease in mortality when compared with patients with CTP class A to C (Fig. 1A & Table 4).

Patients with CTP class 0 were characterized by distinct clinical presentations. These patients had higher prevalence of HBV infection, very well-preserved liver function, lower serum AFP level, better general well-being, smaller tumor burden with more solitary nodule, lower rate of vascular invasion and more frequently treated with curative interventions. It should be noted that advanced CTP classes usually represent a worse residual liver function resulting from chronic inflammation and fibrosis and thus affect the growth and behavior of HCC [25]. Reversely, in patients with large tumors, the large tumor burden may compromise liver functional reserve and have a more advanced cirrhosis stage at presentation. Furthermore, the complications of cirrhosis may influence the general well-being of the patient [26] and the applicability of receiving aggressive treatment for HCC [27]. Importantly, modified CTP classification was not only independently associated with long-term survival; its impact on mortality outweighed most other prognostic factors in the multivariate Cox model after adjusting the confounding effect of treatment strategy. This result strongly implies that modified CTP classification is a powerful and comprehensive indicator in predicting the survival of patients with HCC.

The current tumor staging systems used the original CTP classification in stratification of the tumor stages. After identifying patients with very well-preserved liver function as CTP class 0, the modified CTP classification may be more applicable for tumor staging stratification and treatment allocation. In this regard, we have modified the currently used cancer staging systems for HCC, based on the modified CTP classification, in order to enhance the prognostic ability of these systems. Of all the BCLC-based models, the 3 modified models had better prognostic ability than the original BCLC staging system, indicating that the original system is not informative enough to discriminate the outcome of these HCC patients. In the original BCLC staging systems, the criteria for evaluating liver function are all CTP A–B in patients of stage A, B and C. The same definition for CTP classification would sacrifice a very important parameter in assessing the cancer stages and predicting outcome among patients with original BCLC stage A to C. Reassigning CTP class 0, A, B, B and C to stage 0 to D may enhance the prognostic ability of the original BCLC model and was the most informative one among all modified models. In this modified model, only 2% of patients were selected as stage 0. This group indicates patients with very small tumor size, better liver functional reserve and general well-being, and had 5-year survival rate of 75%. Taken together, the modification of the BCLC system further enhances their prognostic ability in patients with early to advanced cancer stage (Fig. 2 & Table 7).

One of the advantages of the BCLC system is that it can also be used for treatment allocation. Whether the new modified system has a better ability in assigning treatment still needs to be validated in future studies. However, our results imply that the modified BCLC model is more informative in treatment allocation. In the very early stage (stage 0) of the original BCLC system, patients with CTP class A and single tumor nodule with size ≤2 cm are suggested to undergo RFA therapy due to its high complete response rate and good long-term outcome [8], [28]–[31]. However, whether RFA is superior to SR in this group of patients is under intense debate [32]–[34]. In our study, we have further selected patients with very-well preserved liver function for patients receiving RFA. Well preservation of liver function for the applicability of repeated therapy due to tumor recurrence after primary RFA is considered to predict a good long-term survival [35], [36]. Therefore, reassigning CTP class 0 patients to stage 0 may better select candidates with adequate liver functional reserve to receive RFA. Furthermore, in the modified early stage (stage A) of the BCLC model, modified CTP class A replaced the original CTP class A–B for assessing liver function. Surgical resection was considered with a higher risk of mortality among patients with CTP class B [37]–[40] and re-defining the BCLC staging may better select patients for surgical resection.

The CLIP staging system was well validated for its prognostic ability in HCC [4], [41], but early studies disclosed its limitations in discriminating patients with early stage HCC [42]. In our modified CLIP model A, scores of 0–7 were proposed for better discrimination of the patients in terms of outcome prediction. A total of 8% of patients were selected with very-well preserved liver function, small tumor burden and good tumor behavior and predicted a 5-year survival of 81%. The new modified CLIP model showed significant improvements in discriminative ability and prognostic power from very early to advanced cancer stages. Interestingly, consistent with our previous study [41], the modified CLIP model A had the lowest AIC score among all original and modified BCLC, CLIP and TIS staging systems and was considered to be the best prognostic model for outcome prediction in HCC patients. The TIS model was initially proposed to use TTV as the main indicator for tumor burden [6] and its modified model A also showed great discriminatory ability in comparison with the modified BCLC models.

This study has a few limitations. Firstly, HBV is the most common etiology of HCC in Taiwan. This feature is different from most Western countries, where HCV infection and alcoholism are the predominant causes of chronic liver disease. It is our concern whether our results can be applied to patients in the Western countries. Secondly, another important limitation of our study was that the cutoff values for the definition of CTP class 0 were set arbitrary aiming to identify patients with very well-preserved liver function. Thus, validation in future studies is needed. Thirdly, the calculation of TTV for the TIS staging systems was based on the assumption that all tumor nodules were spherical. Therefore, TTV could be slightly overestimated in patients with non-spherical tumor nodules.

In conclusion, the newly proposed CTP classification, aimed to identify patients without overt liver dysfunction as CTP class 0, could accurately predict survival among HCC patients. This proposal is consistently supported by the findings that the modified cancer staging systems of BCLC, CLIP and TIS according to the modified CTP classification have better discriminatory ability and prognostic power when compared with the original models. Treatment strategy according to the modified BCLC model needs to be validated in future studies.
